# Public attitudes toward dairy farm practices and technology related to milk production

**DOI:** 10.1371/journal.pone.0250850

**Published:** 2021-04-30

**Authors:** Lexis H. Ly, Erin B. Ryan, Daniel M. Weary

**Affiliations:** Animal Welfare Program, Faculty of Land and Food Systems, University of British Columbia, Vancouver, BC, Canada; Newcastle University, School of Natural and Environmental Sciences, UNITED KINGDOM

## Abstract

Dairy farm systems have intensified to meet growing demands for animal products, but public opposition to this intensification has also grown due, in part, to concerns about animal welfare. One approach to addressing challenges in agricultural systems has been through the addition of new technologies, including genetic modification. Previous studies have reported some public resistance towards the use of these technologies in agriculture, but this research has assessed public attitudes toward individual practices and technologies and few studies have examined a range of practices on dairy farms. In the present study, we presented participants with four scenarios describing dairy practices (cow-calf separation, the fate of excess dairy calves, pasture access and disbudding). Citizens from Canada and the United States (n = 650) indicated their support (on a 7-point scale) toward five approaches (maintaining standard farm practice, using a naturalistic approach, using a technological approach, or switching to plant-based or yeast-based milk production) aimed at addressing the welfare issues associated with the four dairy practices. Respondents also provided a text-based rationale for their responses and answered a series of demographic questions including age, gender, and diet. Participant diet affected attitudes toward milk alternatives, with vegetarians and vegans showing more support for the plant-based and yeast-based milk production. Regardless of diet, most participants opposed genetic modification technologies and supported more naturalistic practices. Qualitative responses provided insight into participants’ values and concerns, and illustrated a variety of perceived benefits and concerns related to the options presented. Common themes included animal welfare, ethics of animal use, and opposition toward technology. We conclude that Canadian and US citizens consider multiple aspects of farm systems when contemplating animal welfare concerns, and tend to favor naturalistic approaches over technological solutions, especially when the latter are based on genetic modification.

## Introduction

As farm systems have intensified [1] so has public concern due, in part, to perceptions about animal welfare [2, 3]. Studying public attitudes can identify specific animal welfare concerns and thus help inform changes that bring practices in line with public values [4].

One approach to addressing animal welfare-related challenges in agricultural systems is through technological solutions. For example, the challenge of finding suitable labor for milking dairy cows has been addressed by the invention and widespread adoption of automated milking systems; in this case, the technology is also perceived to provide welfare benefits for the cows, for example, by reducing waiting times to be milked and reducing the risk of negative interactions between farm staff and the animals [5].

There is, however, a history of public opposition to some types of technology, perhaps especially when this technology is perceived to be harmful to others, including animals and the environment [6–8]. One relatively modern form of technology, the genetic modification (GM) of plants and animals, provides a topical example. GM animals have been used to improve food quality, productivity, and disease resistance; although few examples to date have entered the market [9]. Previous research has found largely negative public attitudes toward GM animals [6, 10]. Members of the public associate GM animals with “unnaturalness” and often voice ethical concerns over the technology, sometimes relating to animal welfare [11]. Attitudes toward GM are often informed by the perception of risks associated with harming nature, harming animals, and harming human health [12]. Negative attitudes towards GM technology may also be related to feeling that the approach is a “de-natured” solution to these concerns [13].

GM technologies can also be used to address specific animal welfare concerns associated with existing agricultural practices. For example, the painful procedure of disbudding dairy calves (typically achieved by cautery of the horn producing tissue) can be avoided by introducing ‘polled’ (i.e. genetically hornless) genetics via the use of GM [14]. McConnachie et al. [15] found that modifying cows to be hornless was viewed by survey participants as largely positive; however, participants also expressed concerns regarding the perceived unnaturalness of genomic technology. In addition to applications intended to directly address the underlying issue (in this case avoiding the painful procedure of disbudding), technology can be applied to develop alternative products that replace the products of animal agriculture. For example, a variety of plant-based ‘milk’ products have been developed that consumers may prefer for environmental, health, or welfare reasons [16]. These alternative products have seen rapid growth in US markets, while dairy milk consumption has declined [17]. GM technologies have also been applied to the development of milk alternatives; for example, to modify yeast such that they produce milk proteins (creating a type of ‘cowless milk’, as this approach will be referred to henceforth) [18].

Little research has examined public attitudes toward the use of GM technologies that are intended to address ethical concerns associated with farm practices, and previous research has considered individual practices in isolation. The specifics of the practice may affect attitudes, and also limit the types of technology that can be applied to address animal welfare concerns, so it is important to also consider variation in attitudes across practices.

Work to date has found substantial public opposition to dairy farm practices that are perceived to harm animals. For example, cow-calf separation was largely opposed because participants viewed this practice as harmful to cow and calf [19, 20]. Recent concern for the welfare of calves has also emerged due to the practice of selling excess calves shortly after birth [21]. Similarly, research has shown public opposition to disbudding [22] and lack of pasture access [23]. Our study aims to understand attitudes toward technological and other approaches that could be used to address welfare concerns related to cow-calf separation, the fate of excess calves, pasture access and disbudding. In each case, participants were asked to rate their support of the standard practice and of four different approaches to addressing the issue: a naturalistic approach, a technological approach (including ones based upon GM), and rejecting conventional dairy production by switching to plant-based or ‘cowless’ milk.

## Materials and methods

### Participants

Participants were recruited from the US and Canada using Amazon Web Service’s CloudResearch platform. Participants’ quotas were based on representative sampling from the US (2018) and Canadian (2016) census data for age, gender, and income. Participants received various compensation (e.g. gift card, money, reward points), as determined by CloudResearch on the basis of the market panel from which they were sourced. Participants were required to be over the age of 18 to complete the survey. Participants read a brief description of the study and possible risks and then provided written consent prior to the start of the survey. All procedures were approved by the University of British Columbia’s Behavioural Ethics Review Board (Ethics ID: H20-01942).

The total number of participants who began the survey was 1005. Some participants (n = 53) provided one-word answers to multiple open-ended responses (e.g. “okay”, “no”, “nothing”); these participants were removed as their responses were considered difficult to interpret and indicative of low engagement in the survey. An additional 163 participant responses were removed for not completing the survey. A further 106 participant responses were removed for failing the attention check; the attention check used in the study was an Instructed Response Item [24], which asked participants to select a particular Likert number. Median time to completion of the survey was 6 minutes and 54 s. The 33 participants who completed the survey in less than half this time (i.e. less than 3 min and 27 s) were excluded from the analysis. After applying these exclusion criteria, the final sample included 650 participants of which 334 were Canadian. Demographics of the sample are outlined in Table 1. Relative to US [25] and Canada [26] census values, our survey participants were somewhat more likely to be female, to have a household income of less than $25,000, and have at least four years of post-secondary education.

**Table 1:** Demographics of the survey participants (n = 650).

Demographic variable	Percent of respondents
**Gender**	
Male	41.0%
Female	58.0%
**Income**	
Less than $25,000	24.9%
$25,000 - $34,999	11.2%
$35,000 - $49,999	13.1%
$50,000-$74,999	17.1%
$75,000-$99,999	15.2%
$100,000-$149,000	12.0%
$150,000 or more	6.6%
**Age**	
18–29	22.0%
30–44	26.2%
45–59	26.2%
Over 60	25.4%
**Education**	
Less than 4 yrs of post-secondary education	58.8%
At least 4 yrs of post-secondary education	41.2%
**Diet**	
Vegetarian or Vegan	9.1%

All individuals were over 18 years old and sourced through CloudResearch’s Prime Panels service.

### Survey

Following consent, participants completed our online survey hosted on Qualtrics. Each participant was exposed to three of the four scenarios in random order: one of the two scenarios related to calf rearing, one to dehorning, and one to pasture access (see S1 File). Within the calf rearing scenario there were two variants (one regarding the fate excess calves and the other regarding cow-calf separation; these were assigned at random across participants). Only one of the two calf rearing variants were shown, as both described similar practices, although one focused more on the separation of cows and calves, while the other discussed selling excess calves. Survey statements had a Flesch Reading Ease score (FRES) of 60–80, which indicates a plain English readability for grade levels 7–9 [27]. After reading the scenario, participants were asked to describe what they believed to be standard practice on farms followed by a description of the actual farm standard practice (Table 2).

**Table 2:** Background information given to participants for each scenario of the survey.

Survey Wording	Excess Calves	Calf Rear	Horn	Pasture Access
**Background statement**	Consider a jug of milk like you would find at your local grocery store. To produce this milk, dairy cows must give birth to a calf approximately once a year. Some female calves are kept to become dairy cows. To the best of your knowledge, how do farmers normally deal with excess calves (male and female) that are not needed on the farm?	Consider a jug of milk like you would find at your local grocery store. To produce this milk, dairy cows must give birth to a calf approximately once a year. To the best of your knowledge, how do farmers normally deal with these calves?	Consider a jug of milk like you would find at your local grocery store. The dairy cows that produce this milk naturally have horns that can injure other cows and farm workers. To the best of your knowledge, how do farmers normally deal with this issue?	Consider a jug of milk like you would find at your local grocery store. To produce this milk, dairy cows can be kept inside a barn and fed harvested grass and other feeds, or they can be kept on pasture. To the best of your knowledge, how do farmers normally keep their cows?
**Actual**	On most [Canadian/US] dairy farms, any extra female and male calves are sold shortly after birth to be slaughtered or raised as veal or beef	Most [US/Canadian] dairy farms remove the calf from the cow within a few hours of birth; calves are then kept in individual stalls.	Most [US/Canadian] dairy farms use a hot iron to burn the tissue around the horn bud, preventing the development of horns in cows.	Most [US/Canadian] dairy farms provide cows with indoor housing. Cows are kept inside a barn and fed harvested grass and other feeds.

All participants were exposed, in random order, to one of the two calf-related scenarios (Excess Calves and Calf Rear), and to the Horn and Pasture Access scenarios. Participants were shown the “Background statement”, and then shown the current common practice on dairy farms (“Actual”).

After reading the background information, participants were asked to rate their support of the standard practice. In addition, four alternative approaches were rated: one was naturalistic and based upon the principles of organic or biodynamic farming, one was technological (and often based upon the use of GM to address the animal welfare issue), one involved the farm switching to the production of plant-based ‘milk’, and one involved the farm switching to the production of ‘cowless milk’, which uses GM yeast to produce milk proteins (Table 3). Thus, each participant saw a total of five approaches to each scenario. Participants were asked to rate their support for each alternative on a 7-point scale (1 = strongly oppose and 7 = strongly support). Participants then provided an open-ended text response to describe the reasons for their responses to these scenarios. The survey ended with demographic questions including age, gender, diet, and region of residence.

**Table 3:** Five approaches given to participants for each scenario of the survey.

	Excess Calves	Calf Rear	Horn	Pasture Access
**Standard Farm**	Farmers sell extra calves shortly after birth to be slaughtered or raised as veal or beef	Farmers separate the calf and cow shortly after birth; calves are then kept in individual stalls	Farmers use a hot iron to burn the tissue around the horn bud, preventing the development of horns	Farmers keep their cows inside a barn where they are fed harvested grass and other feeds
**Technology**	Farmers treat the cow with genetically modified growth hormone so she produces milk for months longer before needing another calf	Farmers treat the cow with genetically modified growth hormone so she produces milk for months longer before needing another calf	Farmers genetically modify their cows so they are born without horns	Farmers use a computerized gate to let cows choose between outdoor pasture or an indoor barn
**Naturalistic**	Farmers leave the cow and calf together until the calf is weaned and then raised for beef	Farmers leave the cow and calf together until the calf is weaned and then raised for beef	Farmers leave the horns intact but change the way cattle are managed to reduce the risk associated with horns	Farmers keep their cows outside on pasture where they are able to graze
**Plant-based milk**	Farmers stop keeping cows, and instead produce ’plant-based milk’ using cereals, nuts or seeds	Farmers stop keeping cows, and instead produce ’plant-based milk’ using cereals, nuts or seeds	Farmers stop keeping cows, and instead produce ’plant-based milk’ using cereals, nuts or seeds	Farmers stop keeping cows, and instead produce ’plant-based milk’ using cereals, nuts or seeds
**Cowless milk**	Farmers stop keeping cows, and instead produce ’cowless milk’ using genetically modified yeast to make milk proteins	Farmers stop keeping cows, and instead produce ’cowless milk’ using genetically modified yeast to make milk proteins	Farmers stop keeping cows, and instead produce ’cowless milk’ using genetically modified yeast to make milk proteins	Farmers stop keeping cows, and instead produce ’cowless milk’ using genetically modified yeast to make milk proteins

All participants were exposed, in random order, to one of the two calf-related scenarios (Excess Calves and Calf Rear), and to the Horn and Pasture Access scenarios.

### Quantitative analysis

We did not have *a priori* predictions about differences in support based on demographics, and for three of the five approaches (farm standard, technology, naturalistic) wording differently across scenarios. We therefore focused our analysis of demographic effects on the plant-based and cowless milk approaches that were worded identically across all scenarios. The Cronbach’s alpha for support for plant-based and cowless milk was 0.82, indicating that the responses to these two questions could be averaged to create a construct related to support for milk alternatives. Variation in this construct was then used to assess the effect of demographics (country, age, gender, education, income, diet), scenario, and the order in which scenarios were presented. We used a mixed model to account for the clustering of responses within each participant (as each participant responded to multiple scenarios). Our mixed model specified participant ID as a random effect and used compound symmetry as a covariance structure based upon model fit (as determined using AIC).

To assess our main prediction of whether support varied across the five alternatives, we ran separate models for each of the four scenarios considered. The models tested differences in participant support as related to approach (farm standard, technology, naturalistic, plant-based and cowless milk), the order in which these approaches were presented, participant diet, and the interaction between approach and diet. Again, we used a mixed model to account for the clustering of responses within each participant (as each participant responded to each of the different approaches within a scenario), and again our mixed model specified participant ID as a random effect and used compound symmetry as a covariance structure based upon model fit (as determined using AIC).

### Qualitative analysis

Participants were asked open-ended questions for each scenario, but only responses to the first scenario were analyzed (Excess Calves n = 163; Calf Rear n = 157; Horn n = 160; Pasture Access n = 170), as our preliminary analysis indicated that the quantity and quality of open-ended responses declined after the first response, and the content of responses was often repetitive. Following Percy et al. [28], open-ended responses were analyzed using some themes identified in previous literature; these themes (e.g. Animal Welfare, Ethics of Animal Use, Naturalness) were selected from related studies on attitudes toward genetically modified technologies in animals [15, 29]. In addition to the previously identified themes, five other themes (Opposition toward Technology, Importance of Dairy, Impact on Humans, Satisfaction with Dairy System, and Limited Knowledge) were developed through inductive methods. Firstly, one researcher (L. Ly) read through the responses and inductively created an initial codebook. Then, two individuals (L. Ly and M. Blackmore) read and independently deductively coded the first 50 participant responses. These researchers then discussed findings, and edited the codebook based on discrepancies. This process was repeated for four sets of 50 random responses until a final codebook was agreed upon. Then, researchers independently coded 20% of the total available responses according to the previously identified and novel themes. Researchers discussed coding discrepancies and coding was repeated and finalized for this batch of responses. This process was repeated for another 40% of the total responses, with any discrepancies resolved through discussion. Lastly, L. Ly coded the remaining 40% of responses. Initial responses were recoded with the final codebook. Responses could contain multiple themes, and thematic content analysis was used to determine the prevalence of each theme in the dataset [30]. Quotes presented in the results are shown based on best representation of the data.

## Results

### Quantitative results

**Fig 1:**
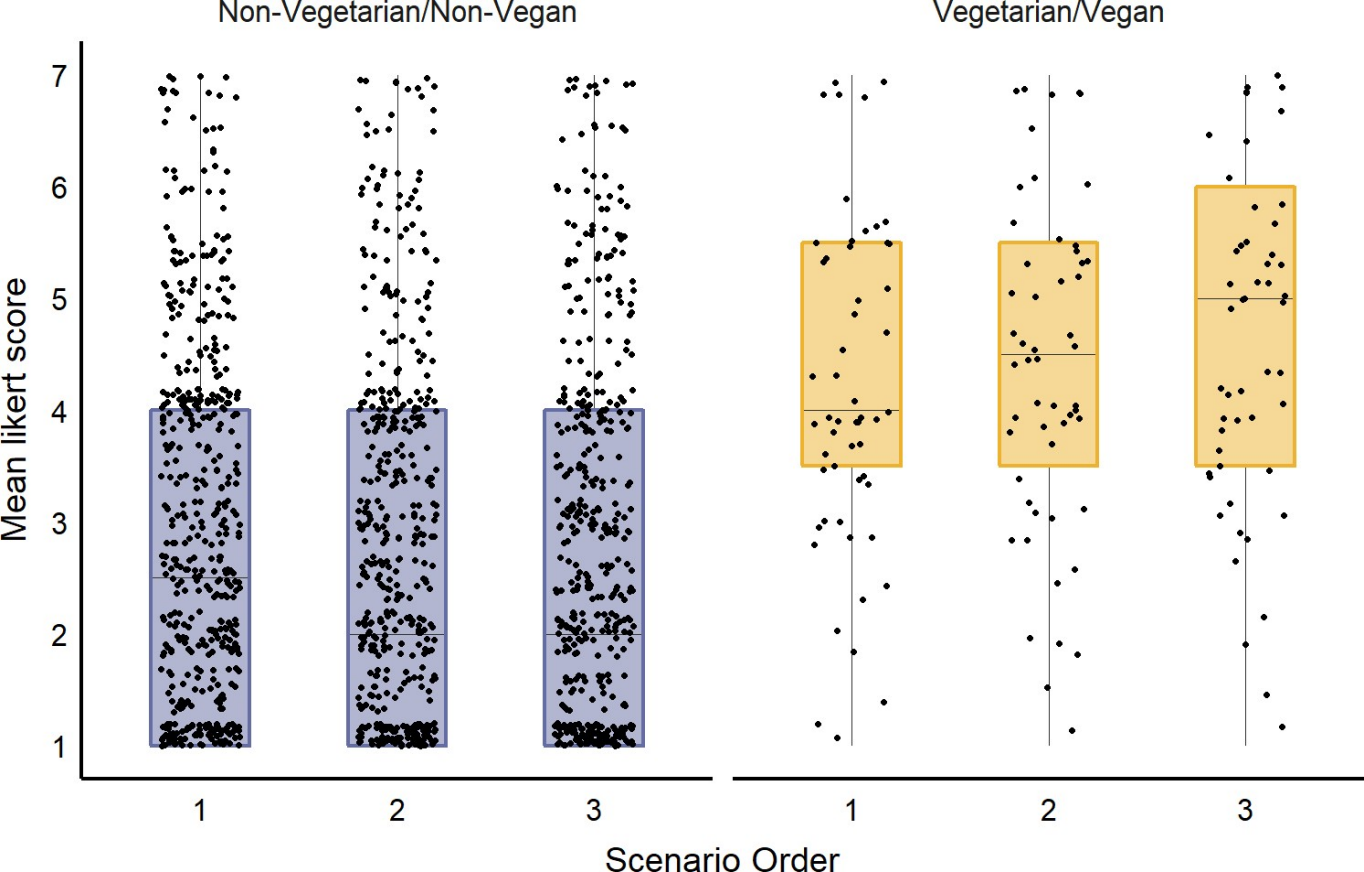
Support for milk alternatives in relation to participant diet and the order in which they encountered treatments. Mean 7-point Likert (where 1 = strongly oppose; 7 = strongly support) averaged across plant-based and cowless milk questions, shown separately for participants who described themselves as vegan or vegetarian (n = 59) and for those who did not (n = 591). Results are also shown in relation to the order that participants encountered the 3 scenarios (one of two calf-rearing treatments, horn removal and pasture access). The central line in the boxplots shows the median, the limits of the box show the 1^st^ and 3^rd^ quartiles, and the whiskers show the 10^th^ and 90^th^ percentiles.

We found no evidence of an effect of country, gender, education, income, or scenario on participant support for the two alternative ‘milk’ products. For every 10-year increase in participant age, mean (± SE) support for alternative ‘milk’ products decreased by 0.3 (± 0.04) on our 7-point Likert (F_1, 642_ = 94.1, p<0.0001). Participants who self-identified as vegetarian or vegan were more supportive of these alternative products, with support averaging 4.3 ± 0.20 on the 7-point Likert, versus just 2.7 ± 0.06 for those who did not describe themselves as vegetarian or vegan (Fig 1; F_1, 642_ = 60.8, p<0.0001). The effect of treatment order varied with participant diet (interaction between question order and diet; F_2,1291_ = 7.38, p = 0.0006). For vegetarian and vegan participants, support increased from 4.2 to 4.5 Likert points over the course of the survey, but for non-vegetarian and non-vegan participants support decreased over the survey from 2.8 to 2.6 Likert points. Given the importance of participant diet on responses to these questions, all results below are analyzed separately by diet.

**Fig 2:**
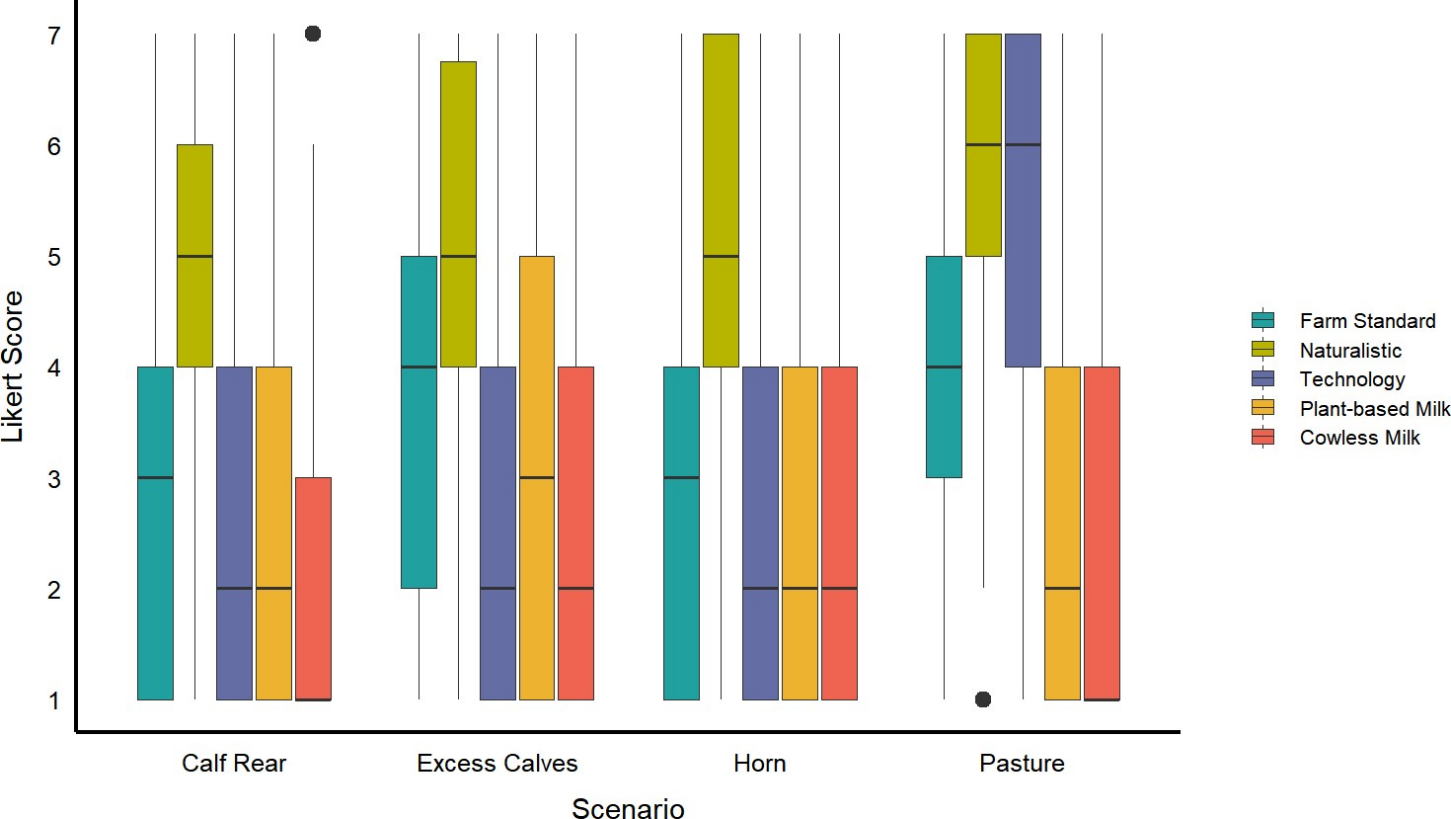
Support of non-vegetarian or vegan respondents for five different approaches to dairy cow welfare issues, shown separately for each of four scenarios (Calf Rear n = 293, Excess Calves n = 298, Horn n = 591, Pasture Access n = 591). Support was expressed on 7-point Likert scale (where 1 = strongly oppose, 7 = strongly support). Participants responded to various approaches to address each presented problem. The central line in the boxplots shows the median, the limits of the box show the 1^st^ and 3^rd^ quartiles, the whiskers show the 10^th^ and 90^th^ percentiles, and the points represent outliers.

Participant responses varied by question and scenario. For participants who did not describe themselves as vegetarian or vegan (Fig 2), there was an effect of question on attitudes for all four scenarios (Calf Rear F_4, 1164_ = 6.85, p<0.0001; Excess Calves F_4, 1180_ = 8.92, p<0.0001; Horn F_4, 2352_ = 29.4, p<0.0001; Pasture Access F_4, 2353_ = 11.1, p<0.0001). The two alternative milk products received low levels of support across all four scenarios, as did the option describing standard farm practice. The technology option was also responded to negatively across all scenarios for which this involved GM; the only technological approach that was responded to favourably was that which described a computerized gating system to provide access to pasture. In contrast, the naturalistic option was responded to positively across all four scenarios.

**Fig 3:**
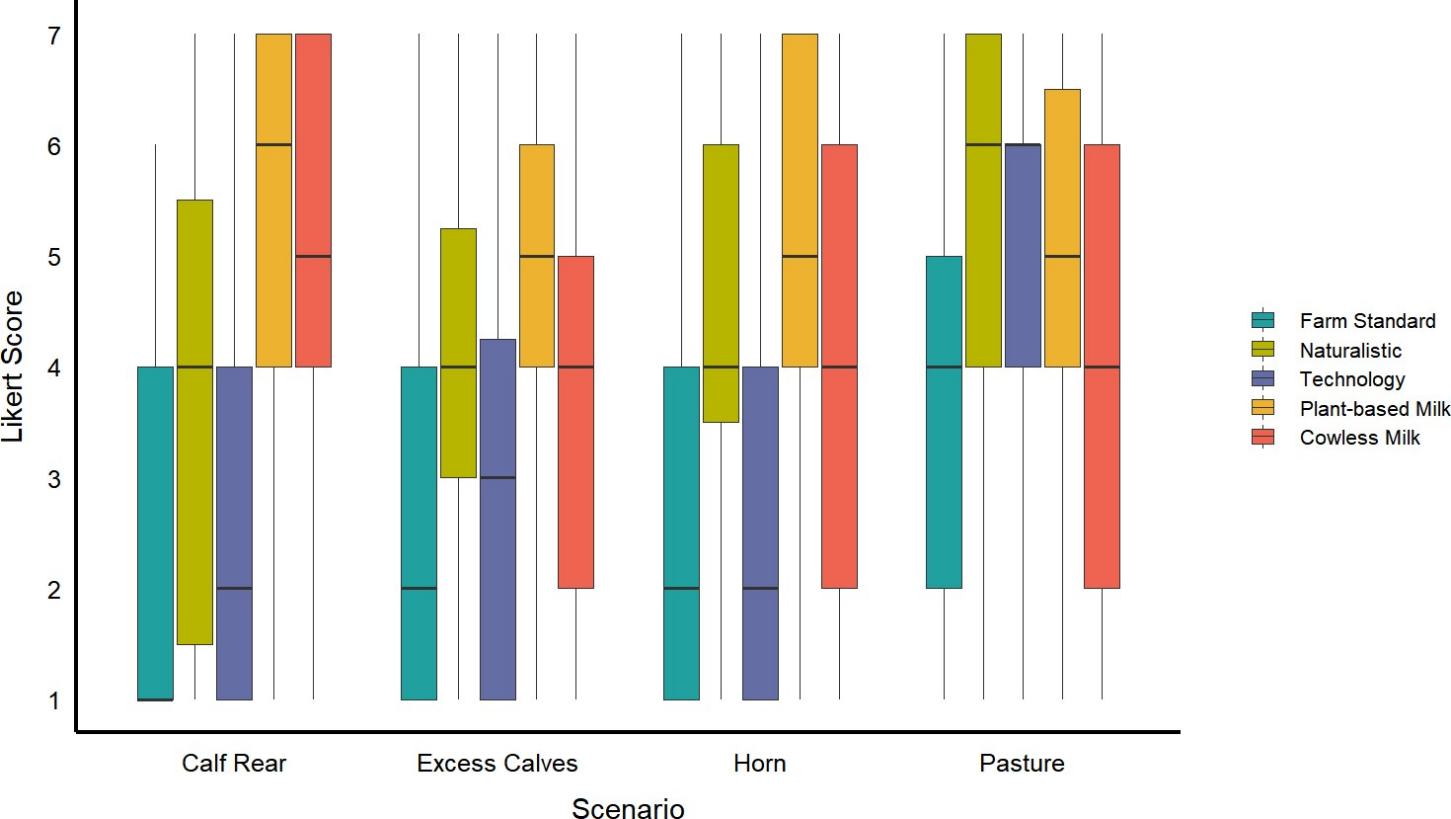
Support of vegetarian or vegan participants for five different approaches to dairy cow welfare issues, shown separately for each of four scenarios (Calf Rear n = 31, Excess n = 28, Horn n = 59, Pasture Access n = 59). Support was expressed on 7-point Likert scale (where 1 = strongly oppose, 7 = strongly support). Participants responded to various approaches to address each presented problem. The central line in the boxplots shows the median, the limits of the box show the 1^st^ and 3^rd^ quartiles, and the whiskers show the 10^th^ and 90^th^ percentiles.

For vegan and vegetarian participants (Fig 3) there was again an effect of question on attitudes across all four scenarios (Calf Rear F_1, 116_ = 9.4, p<0.0001; Excess Calves F_1, 104_ = 0.51 0 = 0.73; Horn F_4, 228_ = 13.2, p<0.0001; Pasture Access F_4, 228_ = 1.14, p<0.0001). The pattern of responses was similar to the non-vegetarians and non-vegans, except for the increased support for the cowless and especially the plant-based milk option; this demographic showed opposition toward standard farm practices and support for the naturalistic solutions much like those who did not describe themselves as vegetarian or vegan.

### Qualitative results

Eight themes were present in the qualitative analysis of the open-ended questions. In order of prevalence, these themes were 1) Animal Welfare, 2) Ethics of Animal Use, 3) Opposition toward Technology, 4) Importance of Dairy, 5) Impact on Humans, 6) Satisfaction with Dairy System, 7) Naturalness, and 8) Limited Knowledge (Table 4). Quotes are cited with participant number and the scenario shown (Calf Rear = CR, Excess Calves = EC, Horn = H, Pasture Access = PA).

**Table 4:** Themes from open-ended responses to the question “please explain your responses above” following the Likert responses regarding support for five different approaches to dairy cow welfare issues.

Theme	Issues discussed in responses	Responses (%)
Animal welfare	Cow or calf’s well-being, quality of life, health, affect states etc.	54.9%
Ethics of animal use	Moral considerations regarding how humans should or should not use and care for animals	35.7%
Opposition toward technology	Dislike, disproval, distrust, or other negative view toward technological approaches	32.3%
Importance of dairy	The value of dairy production, including personal preference and importance of dairy sector for employment	24.8%
Impact on humans	Impacts of consumption of milk or milk alternatives including cost, taste, or human health	22.3%
Satisfaction with dairy system	Contentment with current farming practices or trust in farmers management decisions	19.7%
Naturalness	Natural processes, nature, or the naturalness of dairy practices and alternatives	17.5%
Limited knowledge	Participant stated they lacked adequate knowledge to evaluate options	7.2%

For each theme a brief description is provided. These are worded positively, but responses could have been worded positively or negatively in all cases except “Opposition toward technology”, “Satisfaction with Dairy System” and “Limited knowledge”. Responses could contain more than one theme, so the percentages do not sum to 100.

#### Animal welfare

Responses in the “Animal Welfare” theme discussed quality of life, health, pain, and affective states of cows. While this theme was seen across all scenarios, it was often brought up regarding details of the particular scenario. For example, regarding the Horn scenario, participants often discussed pain associated with disbudding calves (e.g. “it seems painful and cruel to stop their horns from growing” [P618-H]). Participants discussed animal welfare in the Pasture Access scenario in terms of both health and affective states. For example, one participant said, “The cows need to be given the opportunity to roam and get exercise” [P487-PA]. In response to the Pasture Access scenario, another participant said, “I believe the cows are happier and healthier outside” [P270-PA]. Health of calves was also discussed in the Calf Rear scenario (e.g. “They babies need moms milk till they are weaned just like human babies do” [P622-CR]. However, there were some participants who discussed welfare benefits of cow-calf separation, “I think farmers should separate the calves from the mothers not only for the calves’ safety but so the mother can rest” [P830-CR]. Across all themes, there were instances where the cows’ welfare was discussed more broadly (e.g. “The cows should be considered first and their welfare should be the priority” [P425-PA]).

#### Ethics of animal use

Responses coded under this theme described ethical considerations about the use of animals in food production. For example, one participant stated that “Farming animals for milk or meat is inhumane and unnecessary” [P172-PA]. Some participants mentioned the ethics of consuming dairy milk (e.g. “I don’t feel humans should consume cow’s milk. I have always felt cow’s milk should only be consumed by calves” [P662-EC]). Some participants discussed the ethics of animal production as a reason for changing consumption practices (e.g. “I’m all for anything against animal cruelty. I’m vegetarian, trying to go vegan” [P136-PA], “I don’t approve of the way society produces milk. I drink soy and am sticking to it for these reasons” [P56-EC], “I am a pescatarian slowly transitioning into a more plant based diet and lifestyle. I also do not approve of animal cruelty especially for profit” [P52-H]. Others discussed the rights of animals (e.g. “I would want to cows/calves to be treated just like humans” [P77-CR]). Ethical issues were also voiced in relation to the suggested technologies, for example, “Producing milk proteins from modified yeast seems viable to me as a potential solution to the ethics issues involved in raising animals for human consumption” [P379-EC]. Others attempted to balance perceived harms and benefits (e.g. “I believe animals should be treated well but I also know they are a food source” [P526-EC] and “I don’t agree with animal cruelty, but I also believe in the food chain hierarchy” [P600-CR]).

#### Opposition toward technology

This theme captures the qualitative responses that simply reiterated opposition toward one or more of these technologies, with the most common being GM (e.g. “GM [organisms] is a no go for me” [P116-CR]) and cowless milk (e.g. “I don’t support cowless milk at all” [P70-PA]), often without further justification. There was less outright opposition toward plant-based milk, but participants expressed views such as, “I am not in favour of plant based foods and drinks” [P288-H]. Some participants also expressed opposition toward multiple technologies, for example, “I am not a big fan of any of the suggestions especially cowless or ‘plant-based’ milk” [P96-EC]. This theme also included those who qualified their support with any of the other themes. For example, the participant response, “I am opposed to plant based milk. I don’t like the taste and feel they are not as good for the body as natural milk” [P740-EC] was coded under both “Opposition toward technology” and “Impact on humans” themes.

#### Importance of dairy

This theme described various reasons why participants believed the current dairy system was important. This mainly included personal preference (e.g. “I like cow’s milk and wouldn’t want them to stop producing it completely” [P57-CR]); however, participants also discussed societal preference for dairy milk (e.g. “I think milk is an important part of our society” [P229-H]) and the idea that dairy cows are the only “real” source of milk (e.g. “Milk comes from cows and is not made from plants. That would not be milk. It would be a plant drink” [P270-PA]). Respondents in this theme also discussed the importance of dairy farming as a means of employment, for example, “Farmers need to do what is best for them to continue to have a business in dairy” [P528-EC].

#### Impact on humans

This theme covered responses describing impacts in terms of taste, cost, choice, and health. Many concerns were phrased as barriers to consuming alternative products. Regarding cost, one participant said, “I would stop drinking cow milk if there were affordable alternatives” [P286-EC]. Others expressed importance of choice for consumers, for example, “Sure there are people that drink almond or soya milk but it’s not everyone in the population. Some people like drinking milk, so it’s important to have both in store” [P190-EC]. Some participants described taste of milk products being important to them in general, but many discussed negative attitudes about the taste of plant-based milk (e.g. “I haven’t tasted a plant based milk that can match cow’s milk” [P38-PA] and “While I will drink plant-based milk, it just doesn’t taste as good as real dairy milk” [P626-PA]). Participants discussed health benefits and drawbacks across all suggested approaches. Many speculated about the nutrition of milk alternatives (e.g. “Cowless milk is not as nutritional as cow milk” [P947-PA], and “I’m not sure how I feel about farmers using cowless milk because I’m not educated enough on if this method is considered healthy or not” [P745-PA]). Participants also discussed health benefits of these technology-based approaches (e.g. “We must become more plant based for health purposes” [P569-CR]). Some participants also discussed the health benefits of dairy milk (e.g. “Milk is a healthy and beneficial agricultural product” [P708-CR]), while others discussed lack of health benefits of the alternative products (e.g. “Some of [the options] are actually good because cow milk isn’t all good for human health” [P829-H].

#### Satisfaction with dairy system

Responses related to this theme expressed general contentment with the current modern farm system and were all positively worded. There were responses which praised the current dairy system (e.g. “In Canada the farmer takes good care of their animals and there is regulation for it” [P190-EC]) or expressed trust in the dairy farmers’ decisions (e.g. “Farmers know what is best for their business. They should not be told how to run their business based on peoples’ preferences.” [P827-PA]). This theme also included responses which discussed lack of need for change (e.g. “I think it works fine and doesn’t have to change” [P326-EC]) or tradition in dairy farming (e.g. “I believe that there is nothing wrong with the way farmers do their jobs. It has worked for thousands of years, don’t mess with it now” [P696-EC]).

#### Naturalness

Most participants in this theme discussed the naturalness or unnaturalness of the proposed approaches. This theme often emerged in response to technology-based approaches (e.g. “I don’t support cowless milk as it sounds so unnatural” [P81-PA]). However, participants also discussed naturalness in relation to dairy farm management procedures (“The natural way is for cows to graze on pasture” [P636-PA] and “I find the natural approach to raising cows like they do on small farms a better approach” [P284-CR]). Finally, participants commonly appealed to nature as a decision-making approach, for example, “Anything that goes against nature is a bad idea” [P176-EC].

#### Limited knowledge

The limited knowledge theme included references to farm management (e.g. “I don’t know anything at all about farming but my answers are just the way I feel” [P789-EC]) or a particular practice (“Since I don’t know the difference between cows kept in a barn, or allowed to graze outside, I can’t really state which would be better, or which I would support more. This is why I gave the same rating to both methods” [P207-PA] and “I was unaware that farmers sold excess cows to be slaughtered or to be raised as beef” [P96-EC]. Responses often expressed a desire for more information to make a decision (e.g. “I don’t know that ’imitation’ milk is a good replacement for cow’s milk. Would have to see and read information on that” [P744-PA]).

## Discussion

Previous research has investigated public attitudes toward GM [7, 31], farm animal welfare [3, 19], and milk alternatives [32], but to our knowledge no study has considered these issues in combination. The current study provides an exploration of attitudes toward various alternative production methods in relation to the standard of current practice, and does so in the context of a range of contentious issues. Overall, respondents showed more acceptance of naturalistic farming practices and opposition toward GM technology-based options. Vegetarian and vegan participants showed support for plant-based and cowless milk, but these options were largely rejected by other participants. Previous studies that have assessed attitudes to farms more holistically also found that participant values ranged widely [33], consistent with the findings of the current study.

For each of the four scenarios presented, participants showed the most support for the naturalistic alternative. Previous studies have shown that participants support providing a more natural living environment for animals [3, 34], and for practices perceived as being more natural [20, 35]. Schuppli et al. [23] found that 80% of participants agreed with allowing pasture access for cows. Additionally, multiple studies have found that participants favour keeping cows and calves together [19, 36]. These results are consistent with the findings of our study.

The importance of naturalness was also expressed through qualitative responses, where participants discussed unnaturalness of technological solutions, naturalness in relation to management procedures, and naturalness as a basis for decision making. Other research has explored naturalness in relation to food production, revealing some variation in how the term is understood [37]. For example, Jorge et al. [38] surveyed university students regarding food naturalness and found that three factors emerged: how the food is produced (e.g. ingredients, additives, preservatives), how the food is grown (e.g. locality) and trust in the production system (e.g. natural food is better). While GM is not an ingredient or additive, people often believe that GM food does not retain its natural properties [39]. Buller & Morris [13] argued that GM (and indeed the plant-based alternatives) can be seen as “de-natured” approaches to welfare issues.

Participants discussed naturalness as a benefit of “small farms”. Previous research has shown that people sometimes conflate small farms with naturalness [3]; this is discussed in Jorge et al.’s [38] work as an aspect of how food is grown. The present study did not explicitly assess attitudes in relation to the aspect of farm size. The scenarios considered were selected by the authors as reflective of animal welfare concerns in dairy production; we did not ask participants about their concerns regarding dairy production. Other studies have identified other concerns related to modern farming systems that do not pertain to animal welfare, such as how local the farm is [40], or whether the farm is family-owned [3]. Further work may wish to consider a wider range of scenarios, including those that raise issues unrelated to animal welfare, to determine if participants are more receptive of technological solutions in these contexts.

Participants in our study showed at least some opposition to current practices. There was an overlap of themes seen here, as aspects of naturalness were also expressed under the theme “Animal Welfare”, as participants viewed the expression of natural behaviour and access to a more natural living environment as important for welfare. These results are consistent with previous literature showing public concern towards modern practices [3, 41, 42]. More specifically, previous work has identified opposition to lack of pasture access [23], disbudding [35], and cow-calf separation [20]. We compared attitudes across scenarios and found that participants opposed some practices (e.g. disbudding, cow-calf separation) more so than others (e.g. excess calves, lack of pasture access). Boogaard et al. [33] also found that people were more accepting of selling excess cows and more opposed to cow-calf separation.

Vegetarian and vegan participants showed higher acceptance of the plant-based and cowless milk products. Millennial consumers are potential drivers of this market, as they are earlier adopters of new food products [38]. This relationship was illustrated by the association between age and support for alternative products in our study. Consumption of animal products can pose a moral paradox for people [43], as illustrated by comments within the “Ethics of Animal Use” theme. Vegetarians are often more concerned about animal well-being [44] and are more likely to reject all use of animals by humans [45]. The alternative products allow these consumers to avoid dairy products, and thus also animal use. Some respondents also perceived these products as more healthy; milk substitutes are sometimes promoted as a healthy alternative to dairy milk [46]. Although vegetarians and vegans are more likely to recognize the environmental and animal welfare benefits of cultured meat, they are actually less willing to consume it due to negative perceptions such as taste, appeal, price and unnaturalness [47]. Perhaps this difference in attitudes toward milk alternatives versus cultured meat is due to ingredient makeup; the milk alternatives described in the current study were made from plants or derived from yeast, but cultured meat is often created using animal cells and fetal bovine serum. Future research could explore the differences in attitudes toward alternative animal products and other novel food technologies to better understand these nuances.

The overlap of themes in our study illustrates the difficulties that some participants faced in assessing trade-offs relative to these scenarios and alternatives. For example, many participants appeared to value dairy and meat products but also showed concern for both the animals and humans on dairy farms. Previous work has shown that participants often express a range of values when considering animal production systems. For example, one previous study asked participants to describe characteristics of an ideal dairy farm and found that respondents envisioned high animal welfare, as well as high milk quality and low environmental impact [48]. People value safe, low-cost, high quality products, as well as production methods that favor animal and worker welfare [2].

The wide range of responses may have been accentuated by our relatively brief descriptions of the scenarios and alternatives. Participants sometimes stated that they lacked adequate information. Many people have little knowledge of animal production systems and are removed from farming practices [49]. Recent work has shown that people are often unaware of management practices that take place on dairy farms [20]. Some participants in our study expressed the desire for more information on one or more of the suggested approaches, indicating that knowledge was limited regarding both current farming systems and alternative approaches. A lack of familiarity with milk alternatives may have helped drive opposition among the non-vegetarians and non-vegans; previous work has described neophobic responses toward novel food technologies [50].

Participant attitudes may change if given additional information about the proposed approaches, but the additional context would also add to the risk of framing effects. Indeed, even the relatively brief wording provided may have affected attitudes; previous research has shown how information provisioning can impact attitudes toward animal welfare issues [51]. We worded the scenarios to reflect real-life circumstances on North American dairy farms, but future work could examine the effects of positively versus negatively valenced phrasing, and more specifically ask participants what extra information would be helpful.

Our study had other limitations. For example, we recruited a demographically representative sample, but some demographics were over or under-represented in our analysis due to data-cleaning. For example, our sample was female biased compared to census data [25, 26]. Although we tested the effect of demographics on support for milk alternatives and found no effect of various factors, previous work has found that females show greater concern for animal welfare and are less likely to support the use of GM [31, 52, 53]. The present study used samples from Canada and the US—attitudes may differ in other regions.

Public attitudes are subject to social desirability bias, such that responses were likely skewed toward social norms [54]. However, a related study [15] that attempted to control for this bias by also asking participants about the perceived level of the support by “most Americans”, found little difference between this rating and self-reported support. We employed other methods of reducing bias, such as anonymizing the survey, asking participants to evaluate multiple approaches based on each scenario, and providing similar approaches (e.g. plant-based and cowless milk) across each scenario for comparison [55, 56].

The use of multiple scenarios can be considered both a strength and a weakness of the current study. Similarities in responses across scenarios suggest that our results can be seen as relatively robust to context. However, the differences between scenarios required providing different alternatives. Most importantly, the Pasture Access scenario did not have a GM technology approach, but the other three scenarios all used a relevant example of GM technology. To accommodate these differences, our statistical analysis of differences between alternatives was performed separately by scenario. For the milk alternatives, where wording was identical across scenarios, were able to directly assess the context provided by scenario.

## Conclusions

Participant support varied across alternative dairy farm practices, with little support for current standard practices and approaches based upon GM technology, and more support for naturalistic alternatives. Vegan and vegetarian participants were more supportive of milk alternatives including plant-based milk. Participants described multiple reasons for their support of different alternatives, including differences in animal welfare and the value of dairy products. We conclude that alternatives to contentious practices in animal agriculture based upon GM technology are likely to encounter public opposition.

## Supporting information

S1 File Ly et al. survey. Full survey used to assess attitudes toward dairy farm practices and alternatives.
(DOCX)

S2 File Ly et al. SAS code. Statistical analysis used to assess attitudes.
(SAS)

S1 Table Ly et al. table. Summary statistics of support toward dairy farm practices and alternatives separated by diet.
(XLSX)

S1 Dataset Ly et al. survey dataset. Survey data for all Canada and US participants.
(XLSX)

## References

[1] Clay N, Yurco K. Political ecology of milk: Contested futures of a lively food. Geogr Compass. 2020;14. 10.1111/gec3.12497 33209105 PMC7116387

[2] Boogaard BK, Oosting SJ, Bock BB. Defining sustainability as a socio-cultural concept: Citizen panels visiting dairy farms in the Netherlands. 2007 [cited 12 Nov 2020]. 10.1016/j.livsci.2007.11.004

[3] Spooner JM, Schuppli CA, Fraser D. Attitudes of Canadian citizens toward farm animal welfare: A qualitative study. Livest Sci. 2014;163: 150–158. 10.1016/j.livsci.2014.02.011

[4] Vanhonacker F, Verbeke W. Public and consumer policies for higher welfare food products: Challenges and opportunities. Journal of Agricultural and Environmental Ethics. 2014. pp. 153–171. 10.1007/s10806-013-9479-2

[5] Millar KM, Tomkins SM, White RP, Mepham TB. Consumer attitudes to the use of two dairy technologies. Br Food J. 2002;104: 31–44. 10.1108/00070700210418721

[6] Frewer LJ, Coles D, Houdebine LM, Kleter GA. Attitudes towards genetically modified animals in food production. Br Food J. 2014;116: 1291–1313. 10.1108/BFJ-08-2013-0211

[7] Ritter C, Shriver A, McConnachie E, Robbins J, von Keyserlingk MAG, Weary DM. Public attitudes toward genetic modification in dairy cattle. PLoS One. 2019;14: 1–16. 10.1371/journal.pone.0225372 31790436 PMC6886766

[8] Twine R. Animal genomics and ambivalence: A sociology of animal bodies in agricultural biotechnology. Genomics, Soc Policy. 2007;3: 99–117. 10.1186/1746-5354-3-2-99

[9] Vàzquez-Salat N, Houdebine LM. Will GM animals follow the GM plant fate? Transgenic Res. 2013;22: 5–13. 10.1007/s11248-012-9648-5 22987246

[10] Marques MD, Critchley CR, Walshe J. Attitudes to genetically modified food over time: How trust in organizations and the media cycle predict support. Public Underst Sci. 2015;24: 601–618. 10.1177/0963662514542372 25063421

[11] Frewer LJ, Howard C, Shepherd R. Public concerns in the United Kingdom about general and specific applications of genetic engineering: Risk, benefit, and ethics. Sci Technol Hum Values. 1997;22: 98–124. 10.1177/016224399702200105 11654686

[12] Grunert KG. Current issues in the understanding of consumer food choice. Trends in Food Science and Technology. Elsevier; 2002. pp. 275–285. 10.1016/S0924-2244(02)00137-1

[13] Buller H, Morris C. Farm Animal Welfare: A New Repertoire of Nature-Society Relations or Modernism Re-embedded? Sociol Ruralis. 2003;43: 216–237. 10.1111/1467-9523.00242

[14] Mueller MK, Gee NR, Bures RM. Human-animal interaction as a social determinant of health: Descriptive findings from the health and retirement study. BMC Public Health. 2018;18: 1–8. 10.1186/s12889-018-5188-0 29519232 PMC5844080

[15] McConnachie E, Hötzel MJ, Robbins JA, Shriver A, Weary DM, Von Keyserlingk MAG. Public attitudes towards genetically modified polled cattle. PLoS One. 2019;14: 1–15. 10.1371/journal.pone.0216542 31075123 PMC6510451

[16] Fox N, Ward K. Health, ethics and environment: A qualitative study of vegetarian motivations. Appetite. 2008;50: 422–429. 10.1016/j.appet.2007.09.007 17980457

[17] Clay N, Sexton AE, Garnett T, Lorimer J. Palatable disruption: the politics of plant milk. Agric Human Values. 2020;37: 945–962. 10.1007/s10460-020-10022-y 33184529 PMC7644520

[18] Mouat MJ, Prince R. Cultured meat and cowless milk: on making markets for animal-free food. J Cult Econ. 2018;11: 315–329. 10.1080/17530350.2018.1452277

[19] Busch G, Weary DM, Spiller A, von Keyserlingk MAG. American and German attitudes towards cow-calf separation on dairy farms. Olsson IAS, editor. PLoS One. 2017;12: e0174013. 10.1371/journal.pone.0174013 28301604 PMC5354428

[20] Placzek M, Christoph-Schulz I, Barth K. Public attitude towards cow-calf separation and other common practices of calf rearing in dairy farming—a review. Organic Agriculture. Springer; 2020. pp. 1–10. 10.1007/s13165-020-00321-3

[21] Sumner CL, von Keyserlingk MAG. Canadian dairy cattle veterinarian perspectives on calf welfare. J Dairy Sci. 2018;101: 10303–10316. 10.3168/jds.2018-14859 30197138

[22] Robbins JA, Roberts C, Weary DM, Franks B, von Keyserlingk MAG. Factors influencing public support for dairy tie stall housing in the U.S. PLoS One. 2019;14: 1–14. 10.1371/journal.pone.0216544 31063490 PMC6504086

[23] Schuppli CA, Weary DM. Access to pasture for dairy cows: Responses from an online engagement Welfare of non-traditional pets. J Anim Sci. 2014. 10.2527/jas.2014-7725 25261215

[24] Gummer T, Roßmann J, Silber H. Using Instructed Response Items as Attention Checks in Web Surveys: Properties and Implementation. Sociol Methods Res. 2021;50: 238–264. 10.1177/0049124118769083

[25] US Census Bureau. 2018 US Census. 2018. Available: https://www.census.gov/programs-surveys/acs/news/updates/2018.html

[26] Statistics Canada. 2016 Canadian Census. 2016. Available: https://www12.statcan.gc.ca/datasets/index-eng.cfm?Temporal=2016

[27] Flesch R. A new readability yardstick. J Appl Psychol. 1948;32: 221–225. 10.1037/h0057532 18867058

[28] Percy W, Kostere K, Kostere S. Generic qualitative research in psychology. Qual Rep. 2015;20.

[29] Ventura BA, von Keyserlingk MAG, Schuppli CA, Weary DM. Views on contentious practices in dairy farming: The case of early cow-calf separation. J Dairy Sci. 2013;96: 6105–6116. 10.3168/jds.2012-6040 23791487

[30] White MD, Marsh EE. Content analysis: A flexible methodology. Libr Trends. 2006;55: 22–45. 10.1353/lib.2006.0053

[31] Moerbeek H, Casimir G. Gender differences in consumers’ acceptance of genetically modified foods. Int J Consum Stud. 2005;29: 308–318. 10.1111/j.1470-6431.2005.00441.x

[32] McCarthy KS, Parker M, Ameerally A, Drake SL, Drake MA. Drivers of choice for fluid milk versus plant-based alternatives: What are consumer perceptions of fluid milk? J Dairy Sci. 2017;100: 6125–6138. 10.3168/jds.2016-12519 28551193

[33] Boogaard BK, Bock BB, Oosting SJ, Wiskerke JSC, van der Zijpp AJ. Social acceptance of dairy farming: The ambivalence between the two faces of modernity. J Agric Environ Ethics. 2011;24: 259–282. 10.1007/s10806-010-9256-4

[34] Ellis KA, Billington K, McNeil B, McKeegan DEF. Public opinion on UK milk marketing and dairy cow welfare. Anim Welf. 2009;18: 267–282.

[35] Robbins JA, Weary DM, Schuppli CA, Von Keyserlingk MAG. Stakeholder views on treating pain due to dehorning dairy calves. Anim Welf. 2015;24: 399–406. 10.7120/09627286.24.4.399

[36] Hötzel MJ, Cardoso CS, Roslindo A, von Keyserlingk MAG. Citizens’ views on the practices of zero-grazing and cow-calf separation in the dairy industry: Does providing information increase acceptability? J Dairy Sci. 2017;100: 4150–4160. 10.3168/jds.2016-11933 28259414

[37] Chambers E, Tran T, Chambers E. Natural: A $75 billion word with no definition—Why not? J Sens Stud. 2019;34: e12501. 10.1111/joss.12501

[38] Jorge E, Lopez-Valeiras E, Gonzalez-Sanchez MB. The importance given to food naturalness attributes by millennial university students. Sustain. 2020;12. 10.3390/su12020728

[39] Bäckström A, Pirttilä-Backman AM, Tuorila H. Willingness to try new foods as predicted by social representations and attitude and trait scales. Appetite. 2004;43: 75–83. 10.1016/j.appet.2004.03.004 15262020

[40] Weatherell C, Tregear A, Allinson J. In search of the concerned consumer: UK public perceptions of food, farming and buying local. J Rural Stud. 2003;19: 233–244. 10.1016/S0743-0167(02)00083-9

[41] Bennett RM, Blaney RJP. Estimating the benefits of farm animal welfare legislation using the contingent valuation method. 10.1111/j.1574-0862.2003.tb00149.x

[42] Cornish A, Wilson B, Raubenheimer D, McGreevy P. Demographics regarding belief in non-human animal sentience and emotional empathy with animals: A pilot study among attendees of an animal welfare symposium. Animals. 2018;8: 1–11. 10.3390/ani8100174 30287771 PMC6210928

[43] Bastian B, Loughnan S, Haslam N, Radke HRM. Don’t mind meat? the denial of mind to animals used for human consumption. Personal Soc Psychol Bull. 2012;38: 247–256. 10.1177/0146167211424291 21980158

[44] Loughnan S, Haslam N, Bastian B. The role of meat consumption in the denial of moral status and mind to meat animals. Appetite. 2010;55: 156–159. 10.1016/j.appet.2010.05.043 20488214

[45] Broida J, Tingley L, Kimball R, Miele J. Personality Differences between Pro- and Antivivisectionists. Soc Anim. 1993;1: 129–144. 10.1163/156853093X00037

[46] Haas R, Schnepps A, Pichler A, Meixner O. Cow milk versus plant-based milk substitutes: A comparison of product image and motivational structure of consumption. Sustain. 2019;11. 10.3390/su11185046

[47] Wilks M, Phillips CJC, Fielding K, Hornsey MJ. Testing potential psychological predictors of attitudes towards cultured meat. Appetite. 2019;136: 137–145. 10.1016/j.appet.2019.01.027 30731104

[48] Cardoso CS, JosÃ M, Weary DM, Robbins JA, von Keyserlingk MA. Imagining the ideal dairy farm. 2016. 10.3168/jds.2015-9925 26709190

[49] Macnaghten P. Animals in their Nature. Sociology. 2004;38: 533–551. 10.1177/0038038504043217

[50] Siegrist M. Factors influencing public acceptance of innovative food technologies and products. Trends Food Sci Technol. 2008;19: 603–608. 10.1016/j.tifs.2008.01.017

[51] Tuyttens FAM, Vanhonacker F, Langendries K, Aluwé M, Millet S, Bekaert K, et al. Effect of information provisioning on attitude toward surgical castration of male piglets and alternative strategies for avoiding boar taint. Res Vet Sci. 2011;91: 327–332. 10.1016/j.rvsc.2011.01.005 21300388

[52] Bennett B, D’Souza G, Borisova T, Amarasinghe A. Willingness to consume genetically modified foods—the case of fish and seafood. Aquac Econ Manag. 2005;9: 331–345. 10.1080/13657300500234268

[53] Mckendree MGS, Croney CC, Widmar NJO. Effects of demographic factors and information sources on United States consumer perceptions of animal welfare. J Anim Sci. 2014;92: 3161–3173. 10.2527/jas.2014-6874 24962533

[54] Lai Y, Minegishi K, Boaitey AK. Social desirability bias in farm animal welfare preference resesrch. Annu Meet Agric Appl Econ Assoc. 2020. 10.22004/ag.econ.304375

[55] Larson RB. Controlling social desirability bias. Int J Mark Res. 2019;61: 534–547. 10.1177/1470785318805305

[56] Nederhof AJ. Methods of coping with social desirability bias: A review. Eur J Soc Psychol. 1985;15: 263–280. 10.1002/ejsp.2420150303

