# Supplementary material for: Public attitudes toward dairy farm practices and technology related to milk production
Source: PLoS One. 2021 Apr 30;16(4):e0250850. doi: 10.1371/journal.pone.0250850 (PMC8087093; doi:10.1371/journal.pone.0250850)
Supplement: S1 File — Full survey used to assess attitudes toward dairy farm practices and alternatives. (DOCX) [file pone.0250850.s001.docx]

Start of Block: Consent Form

**Consent to participate in:**  Citizens’ attitudes toward various approaches to modern farm animal production issues   Dr. Dan Weary, Professor and NSERC Industrial Research Chair, Faculty of Land and Food Systems, University of British Columbia, dan.weary@ubc.ca    Erin Ryan, graduate student, University of British Columbia, erin.ryan@alumni.ubc.ca   Lexis Ly, undergraduate student, University of British Columbia, lexisly@mail.ubc.ca

**Recruitment** Participation in this project is open to anyone 18 years of age or older. You were recruited to participate in this study using Amazon’s CloudResearch platform.   Study Procedures You will participate in an online survey (approximately 10 minutes long) where you will share your views on public members’ current issues regarding animal production systems. You will be asked a mixture of open-ended and multiple choice questions. This survey should last approximately 10 minutes.

**Risks** During this study you will read some scenarios describing current practices on modern farm systems. Some of the scenarios described in this study may potentially cause discomfort, including some scenarios which may describe medical procedures. All scenarios described in this survey are representative of common farm animal production practices in North America. Should you feel discomfort, you may leave at any point during the survey. Data from your survey will be collected and made publicly available at the time of publishing, after which point you will not be able to withdraw your data. However, only answers to questions will be collected as data and made available. No data on your personal information or identity will be collected (i.e. all data collected will be de-identified).Some of the scenarios described in this study may potentially cause discomfort, including some scenarios which may describe medical procedures. All scenarios described in this survey are representative of common farm animal management practices in North America.

**Confidentiality** The data you provide will be stored in a secure database for a minimum of 5 years and will be both password protected and encrypted. Data will only be accessible to the research team until it is made publicly available at the time of publication. Your confidentiality will be respected.  Information that discloses your identity will not be released without your consent unless required by law.  All documents will be identified only by code number and kept in a locked filing cabinet, or if kept on a computer, in a password protected file. All electronic data will be encrypted and password-protected. You will not be identified by name in any reports of the completed study.

This online survey is hosted by Qualtrics, a service provider contracted by UBC. If you choose to participate in the survey you understand that your responses will be stored in Toronto, Ontario and backed up in Montreal, Quebec. More information about the privacy and security of The Survey Tool can be found at: https://it.ubc.ca/services/teaching-learning-tools/survey-tool/qualtrics-faqs#privacy. The privacy and security policy of the survey service provider Qualtrics can be found at: http://www.qualtrics.com/privacy-statement/.

If you have any questions or concerns about what we are asking of you, please contact one of the co-investigators of this study. Co-investigator names and emails are listed at the top of the first page of this form. If you have any concerns or complaints about your rights as a research participant and/or your experiences while participating in this study, contact the Research Participant Complaint Line in the UBC Office of Research Ethics at 604-822-8598 or if long distance e-mail RSIL@ors.ubc.ca or call toll free 1-877-822-8598.

**Consent** I am at least 18 years of age and I understand my participation in this study is entirely voluntary and that I may choose to quit at any time. I understand that any information I provide will be anonymous and will only be stored and analyzed for the purposes of this research.
**Ethics ID:** H20-01942

- YES, I consent to participate in this study (1)

End of Block: Consent Form

Start of Block: Introduction: surplus bull

Q6
Consider a jug of milk like you would find at your local grocery store.


To produce this milk, dairy cows must give birth to a calf approximately once a year. Some female calves are kept to become dairy cows. To the best of your knowledge, how do farmers normally deal with extra calves (male and female) that are not needed on the farm?

________________________________________________________________

________________________________________________________________

________________________________________________________________

________________________________________________________________

________________________________________________________________

End of Block: Introduction: surplus bull

Start of Block: Actual: surplus bull

Q10 On most [Canadian/US] dairy farms, any extra female and male calves are sold shortly after birth to be slaughtered or raised as veal or beef

End of Block: Actual: surplus bull

Start of Block: Solutions: Surplus bull calves

| 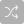 |
| --- |

Q13 How much are you willing to support the following approaches….

|  | 1 (Do not support at all) (1) | 2 (2) | 3 (3) | 4 (Mildly support) (4) | 5 (5) | 6 (6) | 7 (Strongly support) (7) |
| --- | --- | --- | --- | --- | --- | --- | --- |
| Farmers sell extra calves shortly after birth to be slaughtered or raised as veal or beef (1) |  |  |  |  |  |  |  |
| Farmers treat the cow with genetically modified growth hormone so she produces milk for months longer before needing another calf (2) |  |  |  |  |  |  |  |
| Farmers leave the cow and calf together until the calf is weaned and then raised for beef (3) |  |  |  |  |  |  |  |
| Farmers stop keeping cows, and instead produce 'plant-based milk' using cereals, nuts or seeds (4) |  |  |  |  |  |  |  |
| Farmers stop keeping cows, and instead produce 'cowless milk' using genetically modified yeast to make a milk proteins (5) |  |  |  |  |  |  |  |

Q40 Please explain your responses above.

________________________________________________________________

________________________________________________________________

________________________________________________________________

________________________________________________________________

________________________________________________________________

End of Block: Solutions: Surplus bull calves

Start of Block: Introduction: cow-calf

Q5
Consider a jug of milk like you would find at your local grocery store.
 
To produce this milk, dairy cows must give birth to a calf approximately once a year. To the best of your knowledge, how do farmers normally deal with these calves?

________________________________________________________________

________________________________________________________________

________________________________________________________________

________________________________________________________________

________________________________________________________________

End of Block: Introduction: cow-calf

Start of Block: Actual: cow-calf

Q9 Most [Canadian/US] dairy farms remove the calf from the cow within a few hours of birth; calves are then kept in individual stalls.

End of Block: Actual: cow-calf

Start of Block: Solutions Cow Calf

| 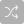 |
| --- |

Q4 How much are you willing to support the following approaches….

|  | 1 (Do not support at all) (1) | 2 (2) | 3 (3) | 4 (Mildly support) (4) | 5 (5) | 6 (6) | 7 (Strongly support) (7) |
| --- | --- | --- | --- | --- | --- | --- | --- |
| Farmers separate the calf and cow shortly after birth; calves are then kept in individual stalls (1) |  |  |  |  |  |  |  |
| Farmers treat the cow with genetically modified growth hormone so she produces milk for months longer before needing another calf (2) |  |  |  |  |  |  |  |
| Farmers leave the cow and calf together until the calf is weaned and then raised for beef (3) |  |  |  |  |  |  |  |
| Farmers stop keeping cows, and instead produce 'plant-based milk' using cereals, nuts or seeds (4) |  |  |  |  |  |  |  |
| Farmers stop keeping cows, and instead produce 'cowless milk' using genetically modified yeast to make a milk proteins (5) |  |  |  |  |  |  |  |

Q41 Please explain your responses above.

________________________________________________________________

________________________________________________________________

________________________________________________________________

________________________________________________________________

________________________________________________________________

End of Block: Solutions Cow Calf

Start of Block: Introduction: horns

Q1
Consider a jug of milk like you would find at your local grocery store.


The dairy cows that produce this milk naturally have horns that can injure other cows and farm workers. To the best of your knowledge, how do farmers normally deal with this issue?

________________________________________________________________

________________________________________________________________

________________________________________________________________

________________________________________________________________

________________________________________________________________

End of Block: Introduction: horns

Start of Block: Actual: horns

Q2 Most [Canadian/US] dairy farms use a hot iron to burn the tissue around the horn bud, preventing the development of horns in cows.

End of Block: Actual: horns

Start of Block: Solutions Horn

| 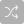 |
| --- |

Q3 How much are you willing to support the following approaches….

|  | 1 (Do not support at all) (1) | 2 (2) | 3 (3) | 4 (Mildly support) (4) | 5 (5) | 6 (6) | 7 (Strongly support) (7) |
| --- | --- | --- | --- | --- | --- | --- | --- |
| Farmers use a hot iron to burn the tissue around the horn bud, preventing the development of horns (1) |  |  |  |  |  |  |  |
| Farmers genetically modify their cows so they are born without horns (2) |  |  |  |  |  |  |  |
| Farmers leave the horns intact but change the way cattle are managed to reduce the risk associated with horns (3) |  |  |  |  |  |  |  |
| Farmers stop keeping cows, and instead produce 'plant-based milk' using cereals, nuts or seeds (4) |  |  |  |  |  |  |  |
| Farmers stop keeping cows, and instead produce 'cowless milk' using genetically modified yeast to make a milk proteins (5) |  |  |  |  |  |  |  |
| To assess if you are paying attention, please select '3' on the scale (7) |  |  |  |  |  |  |  |

Q38 Please explain your responses above.

________________________________________________________________

________________________________________________________________

________________________________________________________________

________________________________________________________________

________________________________________________________________

End of Block: Solutions Horn

Start of Block: Introduction: pasture

Q7
Consider a jug of milk like you would find at your local grocery store.


To produce this milk, dairy cows can be kept inside a barn and fed harvested grass and other feeds, or they can be kept on pasture. To the best of your knowledge, how do farmers normally keep their cows?

________________________________________________________________

________________________________________________________________

________________________________________________________________

________________________________________________________________

________________________________________________________________

End of Block: Introduction: pasture

Start of Block: Actual: pasture

Q11 Most [Canadian/US] dairy farms provide cows with indoor housing. Cows are kept inside a barn and fed harvested grass and other feeds.

End of Block: Actual: pasture

Start of Block: Solutions: Pasture

| 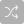 |
| --- |

Q14 How much are you willing to support the following approaches….

|  | 1 (Do not support at all) (1) | 2 (2) | 3 (3) | 4 (Mildly support) (4) | 5 (5) | 6 (6) | 7 (Strongly support) (7) |
| --- | --- | --- | --- | --- | --- | --- | --- |
| Farmers keep their cows inside a barn where they are fed harvested grass and other feeds (1) |  |  |  |  |  |  |  |
| Farmers use a computerized gate to let cows choose between outdoor pasture or an indoor barn (2) |  |  |  |  |  |  |  |
| Farmers keep their cows outside on pasture where they are able to graze (3) |  |  |  |  |  |  |  |
| Farmers stop keeping cows, and instead produce 'plant-based milk' using cereals, nuts or seeds (4) |  |  |  |  |  |  |  |
| Farmers stop keeping cows, and instead produce 'cowless milk' using genetically modified yeast to make a milk proteins (7) |  |  |  |  |  |  |  |

Q39 Please explain your responses above.

________________________________________________________________

________________________________________________________________

________________________________________________________________

________________________________________________________________

________________________________________________________________

End of Block: Solutions: Pasture

Start of Block: Graham et al

| 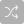 |
| --- |

Q35 When you decide whether something is right or wrong, to what extent are the following considerations relevant to your thinking? Please rate each statement.

|  | Not at all relevant (1) | Not very relevant (2) | Slightly relevant (3) | Somewhat relevant (4) | Very Relevant (5) | Extremely relevant (6) |
| --- | --- | --- | --- | --- | --- | --- |
| Whether or not someone suffered emotionally (1) |  |  |  |  |  |  |
| Whether or not someone cared for someone weak or vulnerable (2) |  |  |  |  |  |  |
| Whether or not someone was cruel (3) |  |  |  |  |  |  |
| Whether or not some people were treated differently than others (4) |  |  |  |  |  |  |
| Whether or not someone acted unfairly (5) |  |  |  |  |  |  |
| Whether or not someone was denied his or her rights (6) |  |  |  |  |  |  |
| Whether or not someone violated standards of purity and decency (7) |  |  |  |  |  |  |
| Whether or not someone did something disgusting (8) |  |  |  |  |  |  |
| Whether or not someone acted in a way God would approve of (9) |  |  |  |  |  |  |

| 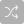 |
| --- |

Q36 Please rate how much you agree with each statement.

|  | Strongly disagree (1) | Moderately disagree (2) | Slightly disagree (3) | Slightly agree (4) | Moderately agree (5) | Strongly agree (6) |
| --- | --- | --- | --- | --- | --- | --- |
| Compassion for those who are suffering is the most crucial virtue (1) |  |  |  |  |  |  |
| One of the worst things a person could do is hurt a defenseless animal (2) |  |  |  |  |  |  |
| It can never be right to kill a human being (3) |  |  |  |  |  |  |
| When the government makes laws, the number one principle should be ensuring that everyone is treated fairly (4) |  |  |  |  |  |  |
| Justice is the most important requirement for a society (5) |  |  |  |  |  |  |
| I think it’s morally wrong that rich children inherit a lot of money while poor children inherit nothing (6) |  |  |  |  |  |  |
| People should not do things that are disgusting, even if no one is harmed (7) |  |  |  |  |  |  |
| I would call some acts wrong on the grounds that they are unnatural (8) |  |  |  |  |  |  |
| Chastity is an important and valuable virtue (9) |  |  |  |  |  |  |

End of Block: Graham et al

Start of Block: Demographics

Q26 What is your age?

________________________________________________________________

Q27 To which gender do you identify?

- Male
- Female
- Other ________________________________________________

Would you describe your diet as any of the following? Check all that apply.

- Vegetarian (do not eat meat)
- Vegan (do not eat meat, fish, or any food derived from animal product)
- Lactose intolerant
- Omnivore (eats food of plant and animal origin)
- Nut allergy
- Soy allergy
- Other (please describe) ________________________________________________

Q49 Which of these products do you consume currently? Check all that apply.

- Dairy (cow) milk
- Plant-based milk (e.g. almond milk, oat milk, soy milk)
- Organic certified milk
- Other milk product (please describe) ________________________________________________
- I do not consume any of these.

What is your highest level of education?

- High school degree or equivalent
- Some university (bachelor's)
- Bachelor's Degree
- Some graduate (MSc, PhD)
- Graduate Degree (MSc, PhD)
- Other (please describe) ________________________________________________

Q50 Which [province/state] do you live in?

End of Block: Demographics
